# Effect of static and dynamic jaw positions on postural stability among people with blindness

**DOI:** 10.1002/brb3.2645

**Published:** 2022-08-02

**Authors:** Ahmad H. Alghadir, Hamayun Zafar, Zaheen Ahmed Iqbal, Shahnawaz Anwer, Amir Iqbal

**Affiliations:** ^1^ Department of Rehabilitation Sciences College of Applied Medical Sciences King Saud University Riyadh Saudi Arabia

**Keywords:** chewing, clenching, jaw functional status, postural stability, tongue position

## Abstract

**Background:**

In comparison with the people with normal sight, the mean center of gravity (COG) velocity is significantly higher among blind people. A strong relationship has been shown between jaw and neck sensorimotor and postural control. The purpose of this study was to determine the effect of different static and dynamic jaw positions on postural stability among subjects with blindness.

**Methods:**

Postural stability was measured as COG velocity in 39 blind subjects under the following five conditions: resting jaw (natural jaw position with no instructions, control), open jaw (teeth of both jaws slightly apart), clenched jaw (teeth tightly closed across each other), chewing (a standard bolus of gum at the natural palace), and tongue position (positioned behind the upper incisors) while standing on firm and foam surfaces.

**Results:**

The mean COG velocity while standing on the firm surface during resting, open jaw position, clenched jaw position, chewing, and tongue positions were 0.54, 0.50, 0.44, 0.59, and 0.46 deg/s, respectively. The mean COG velocity while standing on the foam surface during resting, open jaw position, clenched jaw position, chewing, and tongue positions were 1.42, 1.23, 1.10, 1.14, and 1.06 deg/s, respectively. Compared to the firm surface, the COG velocity was significantly higher on the foam surface in all five conditions (*p* < .001). In the comparison between the conditions, there were no significant differences in either the firm or foam surface in all five conditions (*p* > .05).

**Conclusion:**

People with blindness behave in the same way as sighted subjects on firm and foam surfaces. However, changes in static and dynamic jaw positions do not affect postural stability among them.

## 1 INTRODUCTION

People with visual impairment face difficulties while ambulating and are dependent on their daily activities (Alotaibi et al., 2016; Sadowska et al., 2017). Previous studies among such people show that loss of vision has detrimental effects on postural control (Giagazoglou et al., 2009; Ray & Wolf, 2008). The inability to use visual input prevents one's own visual assessment of body position in space and lowers postural stability (Friedrich et al., 2008; Giagazoglou et al., 2009). They experience serious difficulties while performing motor activities and avoiding obstacles and are at high risk of falling (Brooke‐Wavell et al., 2002; Nakata & Yabe, 2001; Schmid et al., 2007).

To prevent falls, the postural control system decreases the movement of the body through preprogrammed responses that are innate or learned, where somatosensory, visual, vestibular, proprioceptive, cutaneous receptors, and efferent commands provide collective feedback for maintaining balance (Day & Cole, 2002; Gangloff & Perrin, 2002; Kandel, 2000; Keshner, 2003; Rothwell, 2012).

Various studies have indicated a strong connection between the jaw region and postural control (A. Alghadir et al., 2014; A. H. Alghadir et al., 2015a). Various clinical studies show functional, anatomical, biomechanical, neurophysiological, and neuroanatomical relations between the neck and jaw regions (P.‐O. Eriksson et al., 2019). A link between the motor system of the jaw and neck has also been shown by simultaneous movements of the head, neck, and mandible during jaw opening and closing (P.‐O. Eriksson et al., 2007; P. O. Eriksson et al., 1998, 2000; Zafar, Alghadir, & Iqbal, 2019, Zafar et al., 2000, 2002). Changes in the jaw position reorganize the relation between the head, neck, and mandibular region that can further alter the sensory output from high‐density muscle fibers of the region (P. O. Eriksson et al., 2004). These sensations interact with the CNS to reorganize neural settings to control posture through modulation of different reflex systems (P.‐O. Eriksson et al., 2019). The findings also suggest that occlusion can modify posture control in patients with nontraumatic neck pain and whiplash‐associated disorders (P.‐O. Eriksson et al., 2019; Gangloff et al., 2000; Gangloff & Perrin, 2002).

Studies have observed tongue pressure to the palate in patients with cervical pain, and a possible role of the tongue in balance control has been further investigated in healthy young adults (A. H. Alghadir et al., 2015b; P.‐O. Eriksson et al., 2019). These studies seem to note an important connection between various lingual functions, particularly deglutition, and postural control, and confirm that retro incisive spots on the palate could be receptors of the tonic postural system (Ferrante & Scoppa, 2005).

The rationale behind this study was the observations indicating a strong effect of modulation of the jaw and neck sensorimotor system on postural control. Any link between the jaw and postural control systems cannot be proven by investigating healthy subjects alone. We postulated that changes in static and dynamic jaw function can affect balance control among blind subjects. To test this hypothesis, we examined postural sway during quiet standing while resting, open and clenched jaw positions, chewing, and the tongue positioned behind incisors among subjects with blindness.

## 2 METHODS

### 2.1 Subjects

Forty‐five male subjects with visual acuity less than 3/60 in both eyes were invited to participate in this study (Bucci et al., 2009). They were excluded for the presence of any sign of cognitive, balance, jaw, or any skeletal disorder found on examination. After passing this criteria, 39 subjects (mean age 28.8 years, standard deviation 6.86) participated in this study. They were informed about the need for the study, and their written consent was obtained. This study was approved by the institutional ethical review committee for human research in accordance with The Code of Ethics of the World Medical Association (Declaration of Helsinki).

### 2.2 Procedure

Postural stability was measured as the center of gravity (COG) velocity using Neurocom^®^ Balance Master (version 8.5.0, Neurocom International Inc., Clackamas, OR, USA) while standing on a firm and foam surface (A. H. Alghadir, Zafar, et al., 2019; Chien et al., 2007; Liston & Brouwer, 1996; Newstead et al., 2005). For the firm surface, subjects stood on the 46 × 152 cm force platform directly. Foam surface readings were taken by placing a 50 × 50 × 15 cm foam (provided with the balance master) on the force platform. The COG velocity of the natural postural sway was measured as degrees per second (deg/s) and sampled at a frequency of 100 Hz. Balance Master is calibrated automatically during data collection.

The assessment was performed by a physiotherapist who was trained to work with people with visual impairment. Subjects were familiarized with the machine and test procedure prior to the data collection.

### 2.3 Conditions

The COG velocity was recorded in the following five conditions: resting jaw (natural jaw position where no instructions were given, control), open jaw (jaws slightly apart), clenched jaw (jaws tightly closed across each other), chewing (a standard bolus of gum at natural pace), and tongue position (tongue positioned behind the upper incisors) on a firm and foam surface. All recordings were performed in random order. For each condition, there were three trials of 10 s with the rest of approximately 1 min between them. The mean of the three trials was used for analysis.

### 2.4 Statistical analysis

The mean and standard deviation (SD) were used to present descriptive statistics. GraphPad Instat 3.0 software (GraphPad Software, San Diego, CA, USA) was used for statistical analysis. The normality of the data was examined by the Kolmogorov–Smirnov test. Since all the values did not pass the normality test, the difference in COG velocity between the five conditions was tested at the 0.05 level of significance using the Friedman test (nonparametric repeated measures analysis of variance).

## 3 RESULTS

The mean COG velocity while standing on the firm surface during resting, open jaw position, clenched jaw position, chewing, and tongue positions were 0.54, 0.50, 0.44, 0.59, and 0.46 deg/s, respectively. The mean COG velocity while standing on the foam surface during resting, open jaw position, clenched jaw position, chewing, and tongue positions were 1.42, 1.23, 1.10, 1.14, and 1.06 deg/s, respectively (Table 1).

**TABLE 1:** COG velocity (deg/s) in 39 blind subjects during five conditions while standing on firm and foam surfaces: mean (SD)

Condition	Firm	Foam	*p* value
Resting jaw	0.54 (0.31)	1.42 (0.53)	*p* < .001*
Open jaw	0.50 (0.27)	1.23 (0.35)	*p* < .001*
Clenched jaw	0.44 (0.17)	1.10 (0.30)	*p* < .001*
Chewing	0.59 (0.44)	1.14 (0.26)	*p* < .001*
Tongue position	0.46 (0.25)	1.06 (0.27)	*p* < .001*

*Significant value.

In comparison between the two surfaces, there were significantly higher COG velocity values on the foam surface in all five conditions (*p* < .001) (Table 1). In the comparison between the conditions, there were no significant differences in either the firm or foam surface in all five conditions (*p* > .05) (Tables 2 and 3).

**TABLE 2:** Comparison of the COG velocity (deg/s) in 39 blind subjects while standing on a firm surface during five conditions

Condition	Mean (SD)	*p* value
Resting jaw	0.54 (0.31)	*p* > .05
Open jaw	0.50 (0.27)	
Resting jaw	0.54 (0.31)	*p* > .05
Clenched jaw	0.44 (0.17)	
Resting jaw	0.54 (0.31)	*p* > .05
Chewing	0.59 (0.44)	
Resting jaw	0.54 (0.31)	*p* > .05
Tongue position	0.46 (0.25)	
Open jaw	0.50 (0.27)	*p* > .05
Clenched jaw	0.44 (0.17)	
Open jaw	0.50 (0.27)	*p* > .05
Chewing	0.59 (0.44)	
Open jaw	0.50 (0.27)	*p* > .05
Tongue position	0.46 (0.25)	
Clenched jaw	0.44 (0.17)	*p* > .05
Chewing	0.59 (0.44)	
Clenched jaw	0.44 (0.17)	*p* > .05
Tongue position	0.46 (0.25)	
Chewing	0.59 (0.44)	*p* > .05
Tongue position	0.46 (0.25)	

**TABLE 3:** Comparison of the COG velocity (deg/s) in 39 blind subjects while standing on a foam surface

Condition	Mean (SD)	*p* value
Resting jaw	1.42 (0.53)	*p* > .05
Open jaw	1.23 (0.35)	
Resting jaw	1.42 (0.53)	*p* > .05
Clenched jaw	1.10 (0.30)	
Resting jaw	1.42 (0.53)	*p* > .05
Chewing	1.14 (0.26)	
Resting jaw	1.42 (0.53)	*p* > .05
Tongue position	1.06 (0.27)	
Open jaw	1.23 (0.35)	*p* > .05
Clenched jaw	1.10 (0.30)	
Open jaw	1.23 (0.35)	*p* > .05
Chewing	1.14 (0.26)	
Open jaw	1.23 (0.35)	*p* > .05
Tongue position	1.06 (0.27)	
Clenched jaw	1.10 (0.30)	*p* > .05
Chewing	1.14 (0.26)	
Clenched jaw	1.10 (0.30)	*p* > .05
Tongue position	1.06 (0.27)	
Chewing	1.14 (0.26)	*p* > .05
Tongue position	1.06 (0.27)	

## 4 DISCUSSION

This study was performed to determine whether any change in static and dynamic jaw position can affect balance control among subjects with blindness. The results show that compared to a firm surface, the mean COG velocity was higher on the foam surface; however, in the comparison between the conditions, resting jaw, open jaw, clenched jaw, chewing, and tongue position, there were no significant differences on either firm or foam surface. Participants in this study served as their own controls.

The ability of the eye to perceive the shape of objects is called visual acuity. People with impairment of vision have visual acuity less than 6/60 in their best eye; however, visual acuity less than 3/60 is referred to as blindness (Acheson, 2010; De Araújo et al., 2014). The role of vision is important in balance maintenance, and its impairment leads to loss of posture control, neck and shoulder muscle coordination, spinal rotation, and arm swing during gait and an increased likelihood of falling (Portfors‐Yeomans & Riach, 1995; Rosen, 1997). Poor balance results in difficulty in the control of independent navigation (Surakka & Kivelä, 2011). Due to their disability and limited participation in physical activities, people with blindness are at a disadvantage compared to their sighted counterparts (Oh et al., 2004). Balance improvement is important because it can provide the opportunity to walk, run, turn, and jump independently (Keogh & Sugden, 1985). Interventions are needed for the improvement of balance among them (Häkkinen et al., 2006). There are limited studies among people who are visually impaired that assess the risk of falling or analyze potential modifications that can improve postural stability among them.

Postural stability is shown to be reduced during quiet standing and while performing dynamic postural tasks with eyes closed (Buchanan & Horak, 1999; Corna et al., 1999; Dichgans, 1976; Gurfinkel et al., 1976; Schieppati et al., 1999). Larger body sway has been reported in the literature among normal subjects while eyes are closed rather than eyes open (Schieppati et al., 1999). Modification in the reciprocal position of the jaws has been shown to be accompanied by variation in head and neck positions in both sighted and blind individuals (Sforza et al., 2003). The literature about the ability of a blind person to maintain balance in different static and dynamic tasks is either limited or inconclusive. Some studies show that blind subjects can maintain better equilibrium than their sighted counterparts, while other studies show opposite results (Juodžbalienė & Muckus, 2006; Portfors‐Yeomans & Riach, 1995; Pyykkö et al., 1991; Stones & Kozma, 1987). The results of the current study show that postural stability was disturbed among blind subjects while standing on foam surfaces in all five conditions. This confirms that subjects with visual impairment, regardless of eyes open or closed, behave in the same way as sighted subjects with eyes closed (A. H. Alghadir, Alotaibi, et al., 2019; Schmid et al., 2007).

The jaw function is innate and important for the three basic skills of survival: feeding, attack, and defense (Smith, 1999). Similarly, posture and gait control developed with the evolution of human beings (Stedman et al., 2004). A close link between body balance and head‐neck‐jaw position has been observed in healthy subjects (A. Alghadir et al., 2014, 2017; A. H. Alghadir et al., 2015a; Zafar, Alghadir, Iqbal, Iqbal, et al., 2019). Jaw clenching has also been shown to affect the maximal voluntary contraction of limb muscles (A. H. Alghadir, Zafar, et al., 2019). Changes in the jaw motor system have been shown to affect fine motor skills such as handwriting (A. H. Alghadir et al., 2020). However, the results of this study show that there were no significant differences in postural stability in all five jaw positions on both firm and foam surfaces among subjects with visual impairment. This shows that although subjects with blindness behave in the same way as sighted subjects on firm and foam surfaces, changes in static and dynamic jaw positions do not affect their postural stability, as shown in normal subjects.

The tongue is supplied by two motor and four sensory cranial nerves that have musculotendinous connections with the mandible, hyoid, palate epiglottis, and cranium, making it highly sensitive and discriminative (Sicher, 1965; Trulsson & Essick, 1997). While continuously touching the palate, the tongue requires contraction of suprahyoid, infrahyoid, and neck muscles in addition to extrinsic and intrinsic tongue muscles. This thrust reflexively activates jaw‐closing muscles (P.‐O. Eriksson et al., 2019; Miller, 2002). This could be the reason behind enhanced postural control during standing on an unstable surface with eyes closed while the tongue is positioned against the upper incisors in healthy young adults (A. H. Alghadir et al., 2015b). Electrotactile stimulation of the tongue has also been shown to improve postural control during quiet standing, which can be important for enhancing or restoring balance among individuals with compromised systems (Vuillerme et al., 2007). However, the current study did not reproduce similar results among subjects with visual impairment.

The results of the current study differ from the belief that people with blindness have compensatory cross‐modal plasticity and further support the obligatory role of vision in the integration of all sensory inputs in choosing an appropriate body balancing strategy (Schmid et al., 2007). People with compromised sensory systems, such as vision, may use sensory augmentation via various rehabilitation devices, for example, vibrotactile cues, to emphasize the available information from uncompromised systems to improve postural control (Sienko et al., 2018; Umphred et al., 2013). Despite the increasing demand and interest in such techniques, a limited number of researchers have investigated their underlying mechanisms and effectiveness (Bach‐y‐Rita et al., 1969).

## 5 CONCLUSION

People with blindness behave in the same way as sighted subjects on firm and foam surfaces. However, changes in static and dynamic jaw positions do not affect postural stability among them.

## AUTHOR CONTRIBUTION

The research idea and design were proposed by HZ and ZAI. A review of the literature was done by SA and ZAI. Data collection was done by AI and SA. Data analysis was executed by AI and AHA. Project supervision was done by the AHA. Manuscript preparation and submission were done by HZ and ZAI.

## CONFLICT OF INTEREST

The authors declare that they have no competing interests.

### PEER REVIEW

The peer review history for this article is available at https://publons.com/publon/10.1002/brb3.2645.

## Data Availability

The datasets used in this study are available from the corresponding author on request.
